# Excess dietary cholesterol may have an adverse effect on growth performance of early post-larval *Litopenaeus vannamei*

**DOI:** 10.1186/2049-1891-3-19

**Published:** 2012-06-25

**Authors:** Jin Niu, Peng-Fei Chen, Li-Xia Tian, Yong-Jian Liu, Hei-Zhao Lin, Hui-Jun Yang, Gui-Ying Liang

**Affiliations:** 1 Key Laboratory of Aquatic Product Processing, Ministry of Agriculture, South China Sea Fisheries Research Institute, Chinese Academy of Fishery Science, Guangzhou, 510300, People's Republic of China; 2 Nutrition Laboratory, Institute of Aquatic Economic Animals, School of Life Science, Sun Yat-sen University, Guangzhou, 510275, People's Republic of China

**Keywords:** Cholesterol, Growth, Larvae, Lipid classes, *Litopenaeus vannamei*, Survival

## Abstract

One experiment was conducted to determine the nutritive value of cholesterol for post-larval shrimp, *Litopenaeus vannamei*. Four isoenergetic and isonitrogenous diets supplemented with four levels of cholesterol (D1, D2, D3 and D4 with 0, 0.5%, 1% and 2% cholesterol, respectively) were fed to triplicate groups of *L. vannamei* shrimp (mean initial wet weight 0.8 mg) for 27 days. After the trial, shrimp fed the D1 diet had the best growth performance (final body weights: FBW; weight gain: WG; specific growth rate: SGR), while there was no significant difference between diet treatments with respect to survival. The whole body crude protein level in the shrimp decreased with the increase in dietary cholesterol levels, while the whole body crude lipid level in shrimps in the D4 diet treatment was significantly higher (*P* < 0.05) than in other diet treatments. Dietary analysis indicated that the D1 diet contained 0.92% cholesterol prior to supplementation, which may have satisfied the dietary cholesterol requirement of post-larval *L. vannamei*; excess dietary cholesterol may thus lead to adverse effects on the growth performance of post-larval shrimp.

## Background

*Litopenaeus vannamei* is the most common shrimp cultured in the western hemisphere [1] and was introduced into China in 1988. It now is the dominant species in China, mainly cultured in the coastal regions in southern China, but larval shrimp breeding is still dependent on live prey, such as rotifers and *Artemia*. Live prey may be a source of diseases or parasites to the larval rearing system [2]. Furthermore, during the transfer from live prey to artificial diets, high mortality and poor growth of larval shrimp has consistently been observed [3]. The main constraint to the sustainable and healthy development of this species remains the lack of effective and commercially acceptable weaning and on-growing formulated diets. However, substitution of appropriate formulated diets for live prey is crucial for sustaining production of consistently high quality juvenile *L. vannamei*.

An essential step in the development of formulated diet for larval shrimp is to define their nutrient requirements. Cholesterol is an essential precursor of bile acids, steroid hormones, molting hormones, vitamin D_3_ and prostaglandins, which are involved in the molting process in shrimp [4]. Most animals can synthesize sterols from acetate, but crustaceans, like other arthropods, are incapable of *de novo* sterol synthesis from acetate [5]. Therefore, dietary cholesterol is considered essential for good growth and survival of crustaceans. For example, *Penaeus japonicus*[6], larval *P. japonicus*[7], *P. monodon*[8] and *Cherax quadricarinatus*[9] fed a sterol-free/deficient diet had poor growth and survival. However, no research has yet been reported regarding the effects of cholesterol on growth performance of early post-larval *L. vannamei*. Therefore, the objective of the present study was to evaluate whether adding dietary cholesterol could improve the growth performance of early *L. vannamei* post-larvae.

## Materials and methods

### Diet preparation and dietary treatments

Four artificial diets (D1, D2, D3, and D4) were prepared by supplementing cholesterol at 0, 0.5%, 1% and 2% respectively, as shown in Table 1. Cholesterol (95% purity) was purchased from Sigma (Sigma Chemical, St. Louis, MO, USA). Diet analysis indicated that the D1 diet already contained 0.92% cholesterol. The method of diet preparation was the same as described by Niu et al. [9]. Shrimps were acclimatized to the experimental conditions and fed a control diet (D1 without supplemented cholesterol) with a particle size of 300 μm for 3 days before the start of the experiment. The particle size changed to 450 μm, 600 μm, 900 μm and 1.2 mm, from days 1 to 5, 6 to 10, 11 to 21 and 22 to 27 respectively. All diets were stored at −20 °C prior to used.

**Table 1:** Ingredients and proximate composition of experimental diets (% dry matter)

**Ingredients**	**D1**	**D2**	**D3**	**D4**
White fish meal	50.75	50.75	50.75	50.75
Protein hydrolysate	20	20	20	20
a-Starch	5	5	5	5
Soybean oil	3	3	3	3
Phospholipid (purity 97%, pc-60)	2	2	2	2
Vitamin premix^1^	1	1	1	1
Mineral premix^2^	4	4	4	4
Vitamin C	0.65	0.65	0.65	0.65
Krill meal	3	3	3	3
Beer yeast	3	3	3	3
Cellulose	2	1.5	1.0	0
Cholesterol (purity 95%)	0	0.5	1	2
Others^3^	5.6	5.6	5.6	5.6
Proximate composition				
Moisture	7.30	7.27	5.84	5.09
Cholesterol	0.92	1.32	1.80	2.75
Crude protein	57.6	57.8	57.8	57.8
Crude lipid	12.2	12.2	13.0	12.8
Ash	17.0	17.0	17.1	17.0

^1^Contents (g/100 g) retinyl acetate, 0.25; cholecalciferol, 0.625; all-rac-a-tocopheryl acetate, 7.5; menadione, 0.25; thiamin, 0.025; riboflavin, 0.1; D-calcium pantothenate, 0.5; pyridoxine HCL, 0.075; cyanocobalamin, 0.25; niacin, 0.25; folic acid, 0.025; biotine, 0.25; meso-inositol, 37.9; cellulose, 50. (Niu et al. 2008) [10].

^2^Contents (g/100 g) KCL, 9; KI, 4 mg; NaCL, 4; CuSO_4_-5H_2_O, 0.3; ZnSO_4_-7H_2_O, 0.4; CoSO_4_-7H_2_O, 2 mg; FeSO_4_-7H_2_O, 2; MnSO_4_-H_2_O, 0.3; MgSO_4_-7H_2_O, 12.4; Ca(HPO_4_)_2_-2H_2_O, 50; CaCO_3,_ 21.5. (Niu et al. 2008) [10].

^3^Contents (g/100 g): Sodium alginate, 3; Choline chloride, 1; Methionine, 1; Tryptophan, 0.6.

### Experimental system

A 27-day feeding trial was conducted in a recirculating water system. The system was the same as described by Niu et al. [10]. During the trial, the diurnal cycle was 15 h light/9 h dark. Water quality parameters were recorded daily and were maintained as follows: salinity, 30 to 32 g/L; temperature, 27 to 29 °C; dissolved oxygen, 5.6 to 6.2 mg/L; ammonia-nitrogen, 0.05 to 0.07 mg/L.

### Experimental shrimp, feeding and maintenance

The shrimps used were obtained from Evergreen (Zhanjiang) South Ocean Science and Tech Co. Ltd, and the post-larvae were used just after metamorphosis from the mysid stage (15 days post-hatching). Shrimps were collected randomly and groups of 100 shrimps were weighed (following a 24 h fast) before being stocked into individual tanks. Initial average wet weight (0.8 mg) was calculated by dividing the group weight by the number of shrimps. Three replicate tanks (with 1,000 shrimps initially in each tank) were used for each dietary treatment. Shrimps were fed the experimental diets 6 times daily (07:00, 10:00, 13:00, 16:00, 19:00 and 22:00 hours). Feeding quantity was adjusted so that shrimps were fed to slightly to excess. After 27 days of the feeding trial, shrimps were fasted for 24 hours and all surviving shrimps from each tank were weighed as a group. Final average weights were calculated by dividing the group weight by the number of shrimp. Survival was calculated by individually counting all surviving shrimps at the beginning of the experiment and again at the end.

### Sampling and chemical analysis

After weighing, all shrimps in each tank were dried and ground for whole body composition and lipid analysis. Lipids were extracted from the whole body of shrimps with chloroform-methanol [11] and then further separated into neutral lipid and polar lipid fractions by Sep-Pak silica cartridge (Waters, USA) [12]. Both fractions were analyzed for lipid classes using an Iatroscan (MK6, Mitsubishi Chemical Medience, Japan) at the Sun Yat-Sen University of Madical Sciences. Lipid classes were identified by comparison with the appropriate standard (Sigma Chemical, St. Louis, MO, USA). Moisture, crude protein and ash of the experimental diets and shrimps were determined using standard methods of AOAC [13].

### Statistical analysis

All data from triplicate tanks of each diet were analyzed using one-way analysis of variance and Duncan’s multiple-range test. The software was SPSS (Version 10.0). Differences were considered significant at *P* < 0.05.

## Results

### Biological performance of shrimp

Table 2 shows that survival was in the range of 81% to 87%, and no significant difference was found between the groups. Growth performance (FBW, WG and SGR) of shrimp fed the D1 diet was significantly higher than that of shrimp fed the other diets (*P* < 0.05). Moreover, no significant differences were found in growth performance (FBW, WG and SGR) among shrimp fed the D2, D3 and D4 diets (*P* > 0.05).

**Table 2:** Growth performance of shrimp fed a variety of experimental diets

**Cholesterol levels, %**	**D10**	**D20.5**	**D31**	**D42**	**One way ANOVA (*P value*)**
Growth performance					
Initial number	1,000	1,000	1,000	1,000	/
IBW, mg	0.8	0.8	0.8	0.8	/
Final number	865.3 ± 33.5	806.3 ± 49.3	856.7 ± 24.7	853.7 ± 10.7	0.599 (ns)
FBW, mg	53.0 ± 1.5^a^	41.3 ± 0.4^b^	35.3 ± 4.1^b^	33.5 ± 3.8^b^	0.006
WG, %	6529 ± 192^a^	5058 ± 44^b^	4308 ± 514^b^	4092 ± 480^b^	0.006
SGR, %/day	15.5 ± 0.1^a^	14.6 ± 0.0^ab^	14.0 ± 0.4^b^	13.8 ± 0.4^b^	0.015
Survival, %	86.5 ± 3.4	80.6 ± 4.9	85.7 ± 2.5	85.4 ± 1.1	0.599 (ns)

Values are shown as mean ± SE of three replicates. Means within the same row and not sharing a common superscript are significantly different (Ducans, *P* < 0.05); ns, no significant difference detected (*P* > 0.05).

IBW (mg/shrimp): Initial body wet weight (mg/shrimp).

FBW (mg/shrimp): Final body wet weight (mg/shrimp).

WG (%): weight gain = 100 × (final body weight- initial body weight)/initial body weight.

SGR (%/day): specific growth rate = 100 × (ln final wt.- ln initial wt.)/total number of experimental days.

Survival (%) = 100 × (final shrimp number)/(initial shrimp number).

### Whole body lipid class of shrimp

Table 3 shows that total lipid of shrimps fed the D4 diet was significantly higher than that of shrimps fed the other diets, and neutral lipid (NL) had the same tendency as the total lipid. The NL accumulation in whole body of shrimps was (35.1 ± 1.0)%, (36.2 ± 3.0)%, (37.7 ± 1.7)% and (46.6 ± 3.9)% and corresponded with the retention of total cholesterol (TC) at (20.9 ± 0.3)%, (22.4 ± 2.8)%, (23.3 ± 1.3)% and (27.7 ± 3.1)% from the D1, D2, D3 and D4 diet treatments, respectively. The TC accumulation in shrimps fed the D4 diet was significantly higher than that of shrimps in the D1, D2 and D3 dietary treatment groups. The situation for polar lipids (PL) was exactly opposite to the situation for NL. The PL content of shrimps fed the D4 diet was significantly lower (*P* < 0.05) than that of shrimps fed the other diets. The major lipid class of NL fraction was TC, comprising more than 20% of total lipid, while in the PL fraction, phosphatidylcholine (PC) was the main component, comprising approximately 40% of total lipid.

**Table 3:** Total lipid and lipid class of whole body shrimp fed experimental diets

**Cholesterol levels, %**	**D10**	**D20.5**	**D31**	**D42**	**One way ANOVA (*P value*)**
Lipid composition					
Total lipid^1^	1.6 ± 0.1^b^	1.7 ± 0.1^b^	1.7 ± 0.1^b^	2.2 ± 0.1^a^	<0.001
*Neutral lipid*^2^	35.1 ± 1.0^b^	36.2 ± 3.0^b^	37.7 ± 1.7^b^	46.6 ± 3.9^a^	0.008
TC	20.9 ± 0.3^b^	22.4 ± 2.8^b^	23.3 ± 1.3^b^	27.7 ± 3.1^a^	0.043
TG	0.7 ± 0.03	0.6 ± 0.02	0.7 ± 0.04	0.7 ± 0.02	0.986 (ns)
FFA	9.3 ± 0.2^c^	11.7 ± 0.5^b^	14.8 ± 1.1^a^	16.2 ± 0.2^a^	<0.001
*Polar lipid*	64.9 ± 1.1^a^	63.8 ± 3.0^a^	62.3 ± 1.7^a^	53.4 ± 3.9^b^	0.009
PC	40.4 ± 0.5	40.6 ± 1.0	40.1 ± 1.3	37.0 ± 1.4	0.153 (ns)
PE	14.6 ± 0.2^a^	12.5 ± 1.1^ab^	10.7 ± 0.3^b^	8.5 ± 0.6^c^	0.001
PI	0.52 ± 0.02	0.49 ± 0.01	0.53 ± 0.03	0.52 ± 0.05	0.835 (ns)
Others	9.4 ± 0.5	10.2 ± 1.3	11.1 ± 0.6	7.4 ± 2.1	0.311 (ns)

Values are shown as means ± SE of three replicates. Means within the same row and not sharing a common superscript are significantly different (Ducans, *P* < 0.05); ns, no significant difference detected (*P* > 0.05).

TC = total cholesterol; TG = triglycerides; FFA = free fatty acids; PC = phosphatidylcholine; PE = phosphatidylethanolamine; PI = phosphatidylinositol.

^1^% Wet weight.

^2^% Total lipid.

### Whole body composition of shrimp

Table 4 shows that crude protein levels in the whole body of shrimp decreased along with the increase in dietary cholesterol levels, while the crude lipid level in the whole body of shrimp fed the D4 diet was significantly higher (*P* < 0.05) than that of shrimp fed the other diets (D1, D2 and D3).

**Table 4:** Whole body composition (% wet weight) of shrimp fed experimental diets

**Cholesterol levels (%)**	**D10**	**D20.5**	**D31**	**D42**	**One way ANOVA (*P value*)**
Whole body composition					
Moisture	79.7 ± 0.4	80.5 ± 0.6	79.6 ± 0.7	79.7 ± 1.3	0.507 (ns)
Protein	17.9 ± 0.1^a^	17.5 ± 0.3^b^	15.7 ± 0.1^c^	15.2 ± 0.1^d^	<0.001
Lipid	1.6 ± 0.1^b^	1.7 ± 0.1^b^	1.7 ± 0.1^b^	2.2 ± 0.1^a^	<0.001
Ash	3.9 ± 0.1	3.9 ± 0.1	4.0 ± 0.1	4.0 ± 0.1	0.431 (ns)

Values are shown as means ± SE of three replicates. Means within the same row and not sharing a common superscript are significantly different (Ducans, *P* < 0.05); ns, no significant difference detected (*P* > 0.05).

## Discussion

Table 2 shows that the best growth performance (FBW, WG and SGR) of shrimps was found in the D1 diet treatment and the addition of more dietary cholesterol restricted the growth of early *L. vannamei* post-larvae. Dietary composition analysis showed that the basal diet (D1) contained 0.92% cholesterol, which may have satisfied the requirements of early *L. vannamei* post-larvae. This may be due to the use of krill meal as a dietary ingredient, as this is normally a good source of cholesterol. Moreover, due to the cannibalistic nature during the early stages of shrimp development, surviving shrimps may have obtained some cholesterol from consumption of dead shrimps. Sheen et al. [8] reported that diets containing less than 0.8% cholesterol improved growth and survival of *P. monodon*. Thongrod and Boonyaratpalin [14] reported that when the basal diet already contained 0.6% sterol, cholesterol supplementation led to adverse effects, such as retarded growth of banana shrimp, *Penaeus merguiensis*. Sheen [15] reported that mud crabs fed diets containing 0.5% and 0.79% cholesterol had significantly higher weight gain than those fed diets with either lower (0.04% and 0.21%) or higher (1.12% and 1.44%) cholesterol levels, and that cholesterol levels higher than 1.12% had an adverse effect on mud crab growth. Sheen and D’Abramo [16] reported that the level of dietary lipids including phospholipids and cholesterol should be optimum and balanced in order to obtain maximum growth and survival of shrimps, and that high dietary lipid levels may have a detrimental effect on growth performance of crustaceans. Mercer [17] stated that physiological responses to nutrients were graded and produced a characteristic nutrient–response curve, which increased to a point and then tended to level off. The high levels of dietary cholesterol (D2, D3 and D4) which caused the negative growth response in this study may be a nutrient–response characteristic rather than toxicity. The results of this study provide further confirmation that an appropriate dietary cholesterol level is important because high dietary sterol levels may retard growth in crustaceans.

Table 3 shows the concentrations of various classes of lipids in the whole body of shrimps fed diets with and without cholesterol supplementation. The NL accumulation in the whole body of shrimps fed the D4 diet was significantly higher than that of shrimps fed the other diets, and TC accumulation had the same tendency as NL accumulation. This suggests that TC, as the major component of NL was directly influenced by the dietary cholesterol levels, which increased with the increasing amount of dietary cholesterol. Free fatty acids (FFA) accumulation in the whole body of shrimps increased with an increasing amount of dietary cholesterol, although the physiological mechanisms behind this have not been clarified. The situation for PL was exactly the opposite to that of NL. The PL content of shrimps fed the D4 diet was significantly lower than that of shrimps fed the other diets. The major lipid classes of the NL and PL fractions were TC and PC, respectively, and it was TC rather than PC that was influenced by dietary cholesterol levels.

Table 4 shows the whole body composition of shrimps fed diets with and without cholesterol supplementation. The crude lipid content of shrimps fed the D4 diet was significantly higher (*P* < 0.05) than that of shrimp fed the other diets, while the crude protein contents of shrimps decreased with the increase of dietary cholesterol levels. In the study of Sheen [15], both the crude lipid and crude protein levels in the whole body tissue increased with increasing level of dietary cholesterol from 0.21% to 0.79%, then decreased as the level of dietary cholesterol rose to 1.12% and 1.44%. It has been reported that cod larvae may have limited ability to digest neutral lipids [18]. If this is also the case in shrimps, excess addition of dietary cholesterol as neutral lipid may reduce the digestible energy content and lead to an increase in diet consumption in order to use protein as the source of energy. It can therefore be hypothesized that the excess dietary cholesterol was deposited as body lipid, which induced increased dietary protein consumption as a source of energy for growth, but not for body protein deposition.

## Conclusions

In conclusion, the present results show that the level of dietary cholesterol should be strictly controlled; the basal diet already contained 0.92% cholesterol, which may have satisfied the requirement of early *L. vannamei* larvae. Further dietary cholesterol supplementation was detrimental for larval shrimp development.

## Abbreviations

FBW, Final body weight; WG, Weight gain; SGR, Specific growth rate; NL, Neutral lipid; TC, Total cholesterol; PL, Polar lipid; PC, Phosphatidylcholine; FFA, Free fatty acids; PE, Phosphatidylethanolamine; PI, Phosphatidylinositol.

## Competing interests

The authors declare that they have no competing interests.

## Authors’ contributions

JN carried out the experiments and drafted the manuscript. JN and PFC performed the statistical analysis. LXT, HJY HZL and GYL participated in the design of the study. YJL conceived the study, and participated in its design and coordination. All authors read and approved the final manuscript.
